# Inflammasome Signaling in Alzheimer’s Disease after Traumatic Brain Injury

**DOI:** 10.1002/alz.089769

**Published:** 2025-01-03

**Authors:** Juan Pablo de Rivero Vaccari, Erika d.l.R.M Cabrera Ranaldi, Nathan H Johnson, Nadine A Kerr, Helen M Bramlett, Robert W Keane, W Dalton Dietrich

**Affiliations:** ^1^ University of Miami, Miami, FL USA; ^2^ The Miami Project to Cure Paralysis, Miami, FL USA; ^3^ Bruce W. Carter Veterns Affairs Medical Center, Miami, FL USA

## Abstract

**Background:**

The global ageing population is rising with each year, and with that, the percentage of individuals with Alzheimer’s disease (AD) is expected to rise in parallel. Along with age, traumatic brain injury (TBI) is another risk factor for AD. TBI and AD patients demonstrate abnormal inflammatory responses, including that of the inflammasome. The inflammasome is a multi‐protein complex of the innate immune system, that leads to the release of the pro‐inflammatory cytokines interleukin (IL)‐1β and IL‐18 through caspase‐1 and apoptosis‐associated speck‐like protein containing a caspase recruitment domain (ASC). Pyroptosis, a form of inflammatory cell death, also occurs *via* gasdermin‐D cleavage by caspase‐1. Our laboratory has previously demonstrated that genetic predisposition to AD can worsen acute inflammasome activation and outcome after TBI, but the chronic effects of TBI with AD have not been studied.

**Method:**

The 3xTg model of early‐onset familial AD was used, along with their B6129SF2 (WT) controls. AD and WT mice underwent either sham surgery or controlled cortical impact (CCI) and were sacrificed 3‐months post‐injury. Cortical lysates were collected and probed for inflammasome proteins and pro‐inflammatory cytokines via immunoblotting and ECLIAs. Brain sections were utilized for volumetric analysis and immunohistochemistry for inflammasome proteins and degenerative markers.

**Result:**

There was a significant increase in chronic IL‐1β in AD/TBI mice that was not present in WT/TBI mice. Furthermore, there were also increased levels of the inflammasome proteins NLRP3, caspase‐8, and ASC at 3‐months post‐injury in AD/TBI vs WT/TBI mice. AD/TBI demonstrated elevated expression of GFAP, a marker of astrogliosis, which co‐localized with ASC in immunohistochemical analyses. Injured mice showed increased neurofilament light protein, which also co‐localized with ASC. Finally, AD/TBI mice had significant loss of total cortical and hippocampal volume compared to WT/TBI mice.

**Conclusion:**

Our findings establish a chronic dysfunctional inflammatory response in TBI with a genetic predisposition to AD that is not seen in TBI alone. This suggests a unique injury progression that is affected by genetic predisposition to AD that calls for specialized treatment. In addition, due to the increased inflammasome response seen in our results, our conclusions indicate a promising role for the inflammasome as a treatment target.

